# Persistent T-Cell Reactivity in a Seronegative Patient after SARS-CoV-2 Infection and One Vaccination

**DOI:** 10.3390/vaccines10010114

**Published:** 2022-01-13

**Authors:** Nico Andreas, Sebastian Weis, Steffi Kolanos, Sabine Baumgart, Thomas Kamradt, Mathias W. Pletz

**Affiliations:** 1 Institute of Immunology, Jena University Hospital, Friedrich Schiller University, 07743 Jena, Germany; Thomas.Kamradt@med.uni-jena.de; 2 Institute for Infectious Diseases and Infection Control, Jena University Hospital, Friedrich Schiller University, 07747 Jena, Germany; Sebastian.Weis@med.uni-jena.de (S.W.); Steffi.Kolanos@med.uni-jena.de (S.K.); 3 Leibniz Institute for Natural Product Research and Infection Biology-Hans Knöll Institute, 07745 Jena, Germany; 4 Core Facility Cytometry, Institute for Immunology, Jena University Hospital, Friedrich Schiller University, 07743 Jena, Germany; Sabine.Baumgart@med.uni-jena.de

**Keywords:** CoNAN, Neustadt am Rennsteig, COVID-19, SARS-CoV-2, T cell responses, T immunity, antibodies

## Abstract

We present here a 64-year-old male participant of the CoNAN study who experienced a PCR-confirmed mild SARS-CoV-2 infection but did not develop any measurable antibody response. Additionally, after vaccination with ChAdOx1 (AstraZeneca, Cambridge, UK) 11 months later, no antibodies were detected in six serological tests three weeks after the vaccination. When we assessed T-helper (Th) cell immunity, SARS-CoV-2-specific Th cells produced detectable amounts of IFNγ and TNF six weeks after the infection. A robust T-cell immunity remained detectable at least until six months after the infection and was boosted by the vaccination thereafter. This case report points out that an assessment of a prior infection or a vaccine response based solely on antibody detection might have limitations in individual patients.

## 1. Introduction

Common SARS-CoV2-induced COVID-19 symptoms range from nose congestion, cough, sore throat and headache to shortness of breath, diarrhea and loss of smell and taste [1]. Symptom severity of COVID-19 patients has been shown to correlate with age [2], certain comorbidities and sex [3,4]. Th cell responses have been associated with successful eradication of viral infection via induction of IgG production by B cells, cytotoxic T cells, macrophages, and natural killer cells [5,6,7,8,9]. Therefore, considering Th cell responses as an indicator of successful anti-viral immunity might overcome many of the shortcomings of anti-SARS-CoV-2 serology analysis, e.g., timely differences in titers, lack of information about neutralizing efficiency, or individual protective threshold levels [10]. We have previously reported data from the CoNAN study that only 50% of all PCR-confirmed cases were seropositive for anti-SARS-CoV-2 antibodies, with serum levels positively correlating to symptom severity [11]. Conversely, we detected subjects with a prevalence of anti-SARS-CoV-2 serum antibodies without any confirmed PCR result at that early time point of the pandemic [11], suggesting that antibodies would not be a reliable indicator of anti-SARS-CoV-2 immunity. However, cellular mechanisms maintaining immunity to SARS-CoV-2 are not yet fully understood, and a long-term assessment has not yet been possible in large cohorts. Thus, the cellular mechanisms important to achieve long-lasting immunity against COVID-19 are still unclear. 

Here, we report the case of a patient who, despite several risk factors (i.e., sex, age and comorbidities) for severe COVID-19, experienced only a mild disease course. The individual did not develop SARS-CoV-2-specific antibodies after infection nor after subsequent vaccination but showed a robust and long-lasting Th cell response, which was boosted by vaccination.

## 2. Methods

### 2.1. Sample Collection

For antibody measurements, blood was directly centrifuged at 4 °C at 2000× *g* for 10 min and stored at 8 °C thereafter at the study site. For FACS analysis, blood was collected into Na-Heparin tubes and stored at room temperature and in darkness until purification of PBMCs. PBMCs were separated on a Biocoll solution (Bio&SELL GmbH, Feucht, Germany) by centrifugation at 800× *g* at room temperature (RT) for 20 min without brakes. Intermitting phase containing PBMCs was washed with PBS two times and slowly chilled to −80 °C in a Cryo Freezing Container (Thermo Fisher Scientific/Nalgene, MA, USA, rate: −1 °C/min) in a medium containing 10% DMSO (Sigma-Aldrich, Burlington, MA, USA) and 50% FCS (Sigma-Aldrich, Burlington, MA, USA). Subsequently, PBMC samples were cryo-conserved in liquid nitrogen until analysis.

### 2.2. Restimulation of SARS-CoV-2-Specific Th Cells and Cytokine Detection

Cryo-conserved PBMC were thawed at RT and immediately washed with cell culture medium (supplemented with 10% human AB serum (PAN Biotech, Aidenbach, Germany), penicillin/streptomycin). Upon a resting period at 37 °C for 1h, a maximum number of 5 × 10^6^ PBMCs was restimulated in cell culture medium containing 1 µg/mL recombinant anti-human CD28 antibody (clone CD28.2, BioLegend, San Diego, CA, USA, RRID:AB_314303) with either 0.2% DMSO (negative control), SARS-CoV-2 Spike glycoprotein PepMix 1 (S1, N-terminal coverage) or 2 (S2, C-terminal coverage) (both JPT, Berlin, Germany). In total, 10^6^ PBMCs were restimulated with 1 µg/mL TSST1 and 1 µg/mL SEB (both Sigma-Aldrich, Burlington, MA, USA) in the presence of 1 µg/mL recombinant anti-human CD28, or with anti-human CD3/CD28 beads (Gibco/Thermo Fisher Scientific, Vilniaus apskritis, Lithuania) at a ratio of one bead/PBMC as high controls. All samples were incubated for 2 h, and Brefeldin A (BioLegend, San Diego, CA, USA) was added for another 14h of incubation (total 16 h). Upon centrifugation at 300× *g* at RT for 10 min, cells were recovered in 1 mg/mL beriglobin and stained with anti-human CD3 Pacific Blue (clone UCHT1, BioLegend, San Diego, CA, USA, RRID:AB_1595437) and anti-human CD4 Brilliant Violet 605 (clone RPA-T4, BioLegend, San Diego, CA, USA, RRID:AB_2564391). After 5 min, Zombie Aqua fixable dead cells stain (BioLegend, San Diego, CA, USA) was added and incubated for another 10 min. Staining was stopped with PBA/E, and the cells were fixed in 2% Formaldehyde/PBS at RT for 20 min. Intracellular staining with anti-human CD154 APC (clone 24–31, BioLegend, San Diego, CA, USA, RRID:AB_314832), anti-human CD137 PE/Cy7 (clone 4B4-1, BioLegend, San Diego, CA, USA, RRID:AB_2207741), anti-human IFNγ APC/Cy7 (clone 4S.B3, BioLegend, San Diego, CA, USA, AB_10663412) and anti-human TNF PerCP/Cy5.5 (clone MAb11, BioLegend, San Diego, CA, USA, AB_2204081) in 0.5% Saponine (Sigma-Aldrich, Burlington, MA, USA) in PBA/E was performed at 4 °C for 20 min. Upon washing, the cells were recovered in PBA/E and analyzed with a FACS-Canto-Plus flow cytometer (BD, Ashland, OR, USA). Data were analyzed with FlowJo V10.7 (BD, Ashland, OR, USA).

### 2.3. Anti-SARS-CoV-2 IgG Detection

For the serological assessment of anti-SARS-CoV-2 antibodies, the following assays were performed as reported in Weis et al., 2021 [11]: EDI Novel Coronavirus SARS-CoV-2 IgG ELISA kit (Epitope Diagnostics Inc., San Diego, CA, USA) for detecting anti-nucleocapsid antibodies, SARS-CoV-2 IgG ELISA kit (Euroimmun, Lübeck, Germany) for detecting anti-S1 domain antibodies, SARS-CoV-2 S1/S2 IgG CLIA kit (DiaSorin, Saluggia, Italy) for detecting anti-S1/S2 domain antibodies, 2019-nCoV IgG kit (Snibe Co., Ltd., Shenzhen, China) for detecting anti-2019-nCoV recombinant antigen (expressing full-length spike and nucleocapsid proteins) antibodies, SARS-CoV-2 IgG CMIA kit (Abbott, Chicago, IL, USA) for detecting anti-nucleocapsid antibodies and Elecsys Anti-SARS-CoV-2 kit (Roche, Basel, Switzerland) for detecting anti-nucleocapsid antibodies. Sensitivities and specificities were evaluated as described by the manufacturers.

## 3. Case Report and Discussion

During the SARS-CoV-2 outbreak in the Thuringian village of Neustadt am Rennsteig, Germany (described in Weis et al. [11]), in March and April 2020, a 64-year-old male individual with a known history of Diabetes mellitus Type 2, hypertension, chronic heart failure—known risk factors for severe disease course—and prior Hepatitis C infection tested positive for SARS-CoV-2 by PCR obtained by throat swab. In March 2020, the participant developed a sore throat, a congested nose and mild headaches (Table 1). Fever, dyspnea, cough, anosmia or ageusia were not reported. The symptoms lasted approximately three weeks. The patient fully recovered with no indication for Long-COVID or any other sequelae. His concurrent medication was moxonidine, amlodipine, benazepril, metoprolol and empagliflozin.

**Figure 1:**
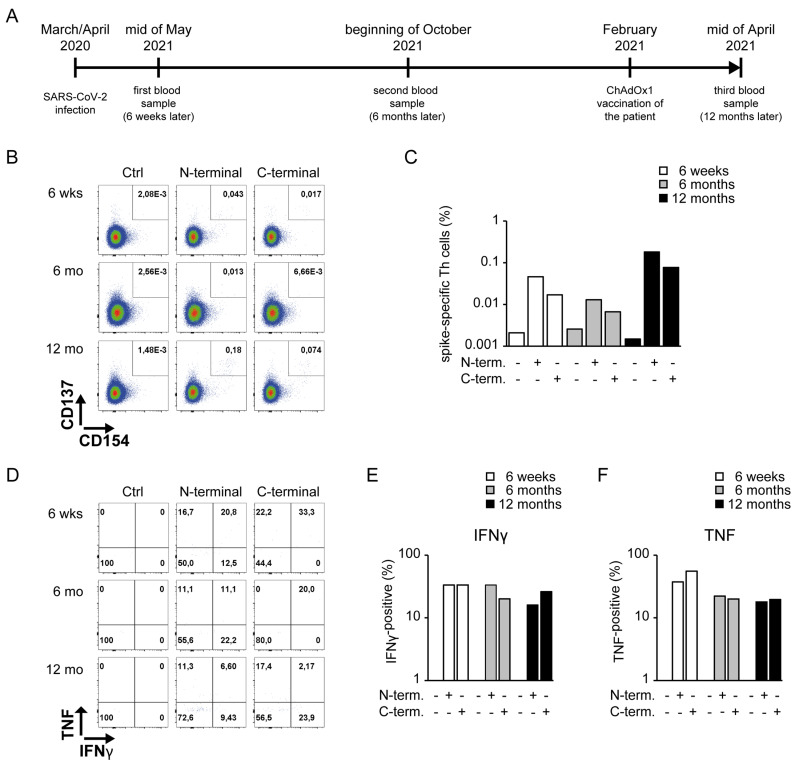
SARS-CoV-2 S protein-specific Th cells in an anti-SARS-CoV-2 antibody-negative patient upon infection and vaccination. Peripheral Blood Mononuclear Cells (PBMCs) collected at several time points were restimulated with peptide mixes covering the SARS-CoV-2 spike protein and cytokine production among SARS-CoV-2-specific Th cells was analyzed. (**A**) Timeline relatively indicates all time points addressed in the case report. (**B**) FACS plots and (**C**) bar plots of antigen-specific CD154^+^CD137^+^ cells among living CD4^+^CD3^+^ Th cells are shown upon indicated restimulation. S-Mix(**N**) indicates SARS-CoV-2 Spike glycoprotein PepMix 1 (N-terminal coverage), and S-Mix (**C**) indicates SARS-CoV-2 Spike glycoprotein PepMix 2 (C-terminal coverage). Presence of a stimulus is indicated with “+” and absence of a stimulus is indicated as “-”. (**D**) FACS plots and (**E**,**F**) bar plots of IFNγ- (**E**) or TNF- (**F**) producing cells among living antigen-specific CD154^+^CD137^+^CD4^+^CD3^+^ Th cells are shown upon indicated restimulation.

He consecutively participated in the CoNAN study in May 2020, a longitudinal cohort study with 626 participants conducted in Neustadt am Rennsteig (Thuringia, Germany) that included an assessment of SARS-CoV-2 nucleic acid (PCR), SARS-CoV-2-specific antibodies (ELISA/chemiluminescence assays) and cellular immunity at six weeks, six months and twelve months after the outbreak [11]. The timeline of all events described in the presented case report is shown in Figure 1A.

A SARS-CoV-2-specific Th cell response can be detected by restimulating PBMCs with peptides of the spike protein, as described by Braun et al. [12]. By flowcytometry, the per-cell expression of Interferon γ (IFNγ) and tumor necrosis factor (TNF) among spike-reactive CD137^+^CD154^+^ double-positive CD4^+^ Th cells was detected (Figure 1B–F). In contrast to the lacking antibody response, a SARS-CoV-2-specific Th cell response (Figure 1B,C) with increased production of IFNγ and TNF (Figure 1D–F) was present. Six months after the infection, none of the conducted six ELISAs/chemiluminescence assays detected any anti-SARS-CoV-2 antibodies, while a SARS-CoV-2-specific Th1 cell response persisted (Figure 1B–F). According to the recommendation of the German Standing Committee on Vaccination (STIKO), the patient received one dose of ChAdOx1 (AstraZeneca, Cambridge, England ) in February 2021, i.e., between the 2nd and 3rd measurement of the CoNAN study. It has been demonstrated that a single vaccination with ChAdOx1 reactivates strong and detectable antibody responses in individuals with previous SARS-CoV-2 infection independent of age [13]. Albeit the antibody induction in elder patients had slower kinetics without previous infection, this study demonstrates the induction of detectable antibody responses in all investigated individuals beyond six weeks after ChAdOx1 vaccination. However, when we re-assessed the serology of this patient during the last CoNAN study visit 12 months after the outbreak and more than 6 weeks after vaccination, again, none of the conducted six ELISAs/chemiluminescence assays detected any antibodies against SARS-CoV-2, but we found a strong and compared to the second blood analysis after six months, even increased SARS-CoV-2-specific Th1 cell response (Figure 1B–F). This indicates that the vaccine did efficiently recall existing Th cell responses in the patient. However, we did not analyze the expression of CD8 in our analysis of CD3^+^CD4^+^ Th cells, and therefore, the reactive T cell subset potentially contained rare circulating human CD4^+^CD8^+^ double-positive T lymphocytes with strong cytokine-producing capacities [14]. We also did not include a B cell lineage-specific marker in our analysis. Of note, at all three measurements, IgM, IgA, IgE, IgG and Ig subclasses were within the normal range, excluding a common variable immune deficiency syndrome or another general dysfunction of antibody production.

## 4. Conclusions

Our data presented in this case report support the relevance of the Th cell response in addition to the antibody-mediated response in SARS-CoV-2 infections [15,16]. Various idiopathic courses of SARS-CoV-2-associated immune responses and COVID-19 have been described, including asymptomatic disease [17], vaccine failure [18,19] or long-term viral shedding [20]. Understanding them might uncover potential mechanisms to successfully prevent severe COVID-19 progression and long-term infections.

The here presented case illustrates that the sole assessment of prior infections and vaccine responses based on antibody detection is not reliable and has serious limitations in individual patients. The efficiency of the cellular response might be directly linked to a reduction in symptoms and could be reactivated by vaccination. Due to the uniqueness of the case, such hypotheses need to be addressed and discussed by further studies.

## Figures and Tables

**Table 1:** PCR test results and reported symptoms.

	March/April 2020	May 2020 (6 Weeks)	October 2020 (6 Months)	April 2021 (1 Year)
PCR tests	1	1	1	1
result	positive	negative	negative	negative
reported symptoms	cold	none	none	none
	sore throat (mild)			
	headache (mild)			

## Data Availability

The data presented in this study are available on request from the corresponding author. The data are not publicly available due to privacy policy.

## References

[1] Dixon B.E., Wools-Kaloustian K.K., Fadel W.F., Duszynski T.J., Yiannoutsos C., Halverson P.K., Menachemi N. (2021). Symptoms and symptom clusters associated with SARS-CoV-2 infection in community-based populations: Results from a statewide epidemiological study. PLoS ONE.

[2] Verity R., Okell L.C., Dorigatti I., Winskill P., Whittaker C., Imai N., Cuomo-Dannenburg G., Thompson H., Walker P.G.T., Fu H. (2020). Estimates of the severity of coronavirus disease 2019: A model-based analysis. Lancet Infect Dis..

[3] Petrilli C.M., Jones S.A., Yang J., Rajagopalan H., O’Donnell L., Chernyak Y., Tobin K.A., Cerfolio R.J., Francois F., Horwitz L.I. (2020). Factors associated with hospital admission and critical illness among 5279 people with coronavirus disease 2019 in New York City: Prospective cohort study. BMJ.

[4] Williamson E.J., Walker A.J., Bhaskaran K., Bacon S., Bates C., Morton C.E., Curtis H.J., Mehrkar A., Evans D., Inglesby P. (2020). Factors associated with COVID-19-related death using OpenSAFELY. Nature.

[5] Guihot A., Luyt C.E., Parrot A., Rousset D., Cavaillon J.M., Boutolleau D., Fitting C., Pajanirassa P., Mallet A., Fartoukh M. (2014). Low titers of serum antibodies inhibiting hemagglutination predict fatal fulminant influenza A(H1N1) 2009 infection. Am. J. Respir. Crit. Care Med..

[6] Zheng M., Gao Y., Wang G., Song G., Liu S., Sun D., Xu Y., Tian Z. (2020). Functional exhaustion of antiviral lymphocytes in COVID-19 patients. Cell Mol. Immunol..

[7] Lee J.S., Koh J.Y., Yi K., Kim Y.I., Park S.J., Kim E.H., Kim S.M., Park S.H., Ju Y.S., Choi Y.K. (2021). Single-cell transcriptome of bronchoalveolar lavage fluid reveals sequential change of macrophages during SARS-CoV-2 infection in ferrets. Nat. Commun..

[8] Zheng H.Y., Zhang M., Yang C.X., Zhang N., Wang X.C., Yang X.P., Dong X.Q., Zheng Y.T. (2020). Elevated exhaustion levels and reduced functional diversity of T cells in peripheral blood may predict severe progression in COVID-19 patients. Cell Mol. Immunol..

[9] Sette A., Crotty S. (2021). Adaptive immunity to SARS-CoV-2 and COVID-19. Cell.

[10] Lippi G., Henry B.M., Plebani M. (2021). Anti-SARS-CoV-2 Antibodies Testing in Recipients of COVID-19 Vaccination: Why, When, and How?. Diagnostics.

[11] Weis S., Scherag A., Baier M., Kiehntopf M., Kamradt T., Kolanos S., Ankert J., Glöckner S., Makarewicz O., Hagel S. (2021). Antibody response using six different serological assays in a completely PCR-tested community after a coronavirus disease 2019 outbreak-the CoNAN study. Clin. Microbiol Infect..

[12] Braun J., Loyal L., Frentsch M., Wendisch D., Georg P., Kurth F., Hippenstiel F., Dingeldey M., Kruse B., Fauchere F. (2020). SARS-CoV-2-reactive T cells in healthy donors and patients with COVID-19. Nature.

[13] Tut G., Lancaster T., Krutikov M., Sylla P., Bone D., Kaur N., Spalkova E., Bentley C., Amin U., Jadir A.T. (2021). Profile of humoral and cellular immune responses to single doses of BNT162b2 or ChAdOx1 nCoV-19 vaccines in residents and staff within residential care homes (VIVALDI): An observational study. Lancet Healthy Longev..

[14] Clenet M.L., Gagnon F., Moratalla A.C., Viel E.C., Arbour N. (2017). Peripheral human CD4(+)CD8(+) T lymphocytes exhibit a memory phenotype and enhanced responses to IL-2, IL-7 and IL-15. Sci. Rep..

[15] Bilich T., Nelde A., Heitmann J.S., Maringer Y., Roerden M., Bauer J., Rieth J., Wacker M., Peter A., Hörber A. (2021). T cell and antibody kinetics delineate SARS-CoV-2 peptides mediating long-term immune responses in COVID-19 convalescent individuals. Sci. Transl. Med..

[16] Bonifacius A., Tischer-Zimmermann S., Dragon A.C., Gussarow D., Vogel A., Krettek U., Gödecke N., Yilmaz M., Kraft A.R.M., Hoeper M.M. (2021). COVID-19 immune signatures reveal stable antiviral T cell function despite declining humoral responses. Immunity.

[17] Oran D.P., Topol E.J. (2021). The Proportion of SARS-CoV-2 Infections That Are Asymptomatic: A Systematic Review. Ann. Intern. Med..

[18] Rincon-Arevalo H., Choi M., Stefanski A.L., Halleck F., Weber U., Szelinski F., Jahrsdörfer B., Schrezenmeier H., Ludwig C., Sattler A. (2021). Impaired humoral immunity to SARS-CoV-2 BNT162b2 vaccine in kidney transplant recipients and dialysis patients. Sci. Immunol..

[19] Collier D.A., Ferreira I., Kotagiri P., Datir R.P., Lim E.Y., Touizer E., Meng B., Abdullahi A., Elmer A., The CITIID-NIHR BioResource COVID-19 Collaboration (2021). Age-related immune response heterogeneity to SARS-CoV-2 vaccine BNT162b2. Nature.

[20] Tarhini H., Recoing A., Bridier-Nahmias A., Rahi M., Lambert C., Martres P., Lucet J.C., Rioux C., Bouzid D., Lebourgeois S. (2021). Long-Term Severe Acute Respiratory Syndrome Coronavirus 2 (SARS-CoV-2) Infectiousness Among Three Immunocompromised Patients: From Prolonged Viral Shedding to SARS-CoV-2 Superinfection. J. Infect. Dis..

